# Oculo-Auriculo-Vertebral Dysplasia With Craniocervical Instability and Occult Tethered Cord Syndrome. An Addition to the Spectrum? First Case Report and Review of the Literature

**DOI:** 10.5435/JAAOSGlobal-D-17-00085

**Published:** 2019-07-30

**Authors:** Nils Hansen-Algenstaedt, Melanie Liem, Salah Khalifah, Alf Giese, Angelika Gutenberg

**Affiliations:** From the Department of Orthopedics, University Medical Center Hamburg Eppendorf, Hamburg, Germany (Dr. Hansen-Algenstaedt); the Department of Spine Surgery, Clinic Manhagen, Grosshansdorf, Germany (Dr. Hansen-Algenstaedt, Dr. Liem, Dr. Khalifah, and Dr. Giese); the Department of Neurosurgery, University Medical Center, Mainz, Germany (Dr. Giese and Dr. Gutenberg); and the Neuroscience Center, King Abdullah Medical City, Makkah, Saudi Arabia (Dr. Khalifah).

## Abstract

Oculo-auriculo-vertebral spectrum (OAVS) is an uncommon congenital disorder of abnormal development of the first and second pharyngeal arches. This spectrum is characterized by craniofacial microsomia, epibulbar dermoids, ear abnormalities, renal and cardiac defects, and a wide range of vertebral segmentation and formation disorders. Frequently, the cervicothoracic spine is involved. Only recently, the morbidity attributed to the spinal abnormalities has gained attention. Strategy and timing of spine surgery has become increasingly important in patients with OAVS. Here, we report a case of OAVS with characteristic vertebral cervical and thoracic involvement and its sequelae requiring multiple spinal procedures, further complexed by an unprecedented occult tethered cord syndrome, which was successfully treated by surgical detethering. In this context, the recent literature on spinal anomalies is reviewed.

First reported by Carl Ferdinand von Arlt in 1845, Maurice Goldenhar^1^ in 1952 described a patient with accessory tragi, ocular dermoids, and mandibular hypoplasia. Gorlin et al^2^ in 1963 named this syndrome oculo-auriculo-vertebral dysplasia, today summarized under oculo-auriculo-vertebral spectrum (OAVS). This condition is a disorder of craniofacial morphogenesis, primarily involving structures derived from the first and second pharyngeal arches.^3^ Symptoms and the physical features may vary greatly in range and severity from case to case, but most often are associated with hemifacial microsomia, micrognathia, cardiac and renal defects, and skeletal and central nervous system (CNS) abnormalities.^3,4,5,6,7,8^ Severe OAVS has a reported incidence of 1 in 27,472 births.^9^

Originally focused on the craniofacial dysmorphic features, more recently, the aspect of the spinal and CNS abnormalities has drawn attention, which eventually may lead to notable morbidity and disability.

## Case History and Treatment

**Figure 1:**
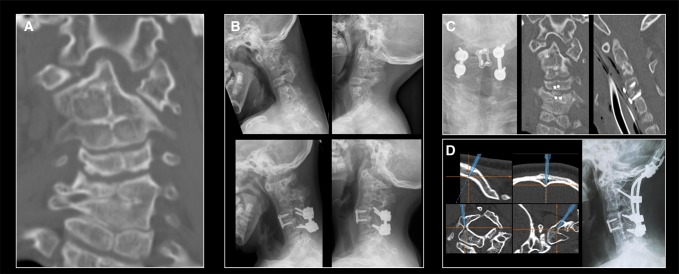
Malfusion of the anterior and posterior arches of C1 and fusion of cervical vertebrae C2/3 and C5/6/7 (**A**) with horizontal instability at C4/5 (**B**, upper panel) in a 7-year-old girl. ACDF C4/5 with dorsal spondylodesis was performed, which resulted in excellent control of symptoms (**C**). At the age of 12 years, C1/C2 horizontal instability increased (**B**, lower panel), and navigation-guided C0-C6 spondylodesis was performed (**D**).

**Figure 2:**
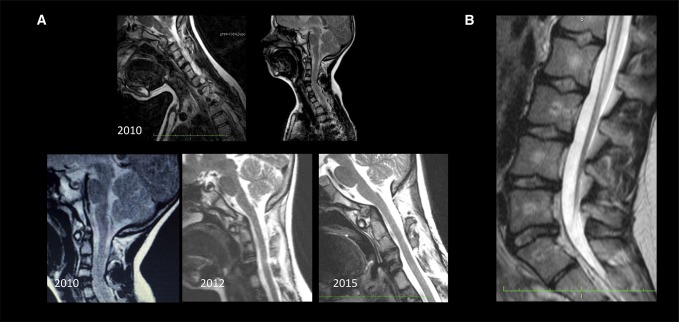
MRI of the cervical spine demonstrating the instability at C4/5 (**A**, upper panel) and the progressive horizontal instability at C1/2 over a period of 5 years (**A**, lower panel). The conus medullaris is located at the level of L1 (**B**).

**Figure 3:**
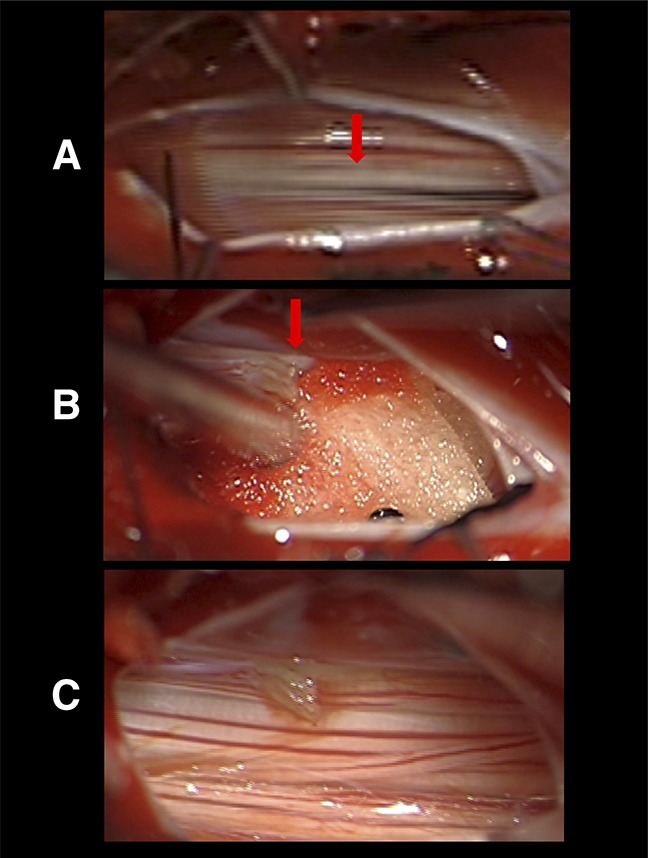
Intradural microscopic view of the tight FT at the L4/5 level. The FT is marked with an arrow, (**A**) before and (**B**) after sectioning. Note the stretching of the cauda equine fibers in (**A**) compared with the relaxed fibers in (**C**). FT = filum terminale

Routine ultrasonography examinations had revealed cardiac abnormalities during the last trimester of pregnancy of a healthy mother with no family history of hereditary diseases or syndromes. The female patient was born with ventricular septal defect (VSD), atrial septal defect (ASD) type II, and stenosis of the pulmonary artery, which at 6 weeks was surgically corrected. Furthermore, facial asymmetry, an epibulbar dermoid, and auricular appendices, but normal ear shapes and hearing, with no ocular abnormalities, were diagnosed. No abnormal chromosomal findings were established. At the age of 3 years, the patient developed nocturnal neck pain and dysesthesia of the lower extremities. The mother noted increasing clumsiness and, as the girl had already completed toilet training, recurring episodes of urinary incontinence during the day. Costovertebral dysplasia of the upper thoracic spine, malfusion of the anterior and posterior arches of C1, and fusion of cervical vertebrae C2/3 and C5/6/7 were found, resulting in kyphotic spinal stenosis and horizontal instability at C4/5 (Figure 1, A). Balanced scoliosis (Cobb angle 36°) of the cervicothoracic spine with a T3 hemivertebra was evident. Conservative treatment with pain medication and orthesis did not sufficiently control neck pain. At the age of 7 years, anterior cervical discectomy and fusion (ACDF) C4/5 with dorsal spondylodesis was performed, which resulted in excellent pain control and no further neurologic symptoms (Figure 1, B and C). However, 4 years later, the patient developed low back pain radiating to both legs and dysesthesia of both feet with episodes of acute deterioration and intermittent urinary incontinence. An MRI showed the conus medullaris at L1, no herniation of the cerebellar tonsils, but a slightly prominent filum terminale (FT) (Figure 2). No other abnormalities of the spinal cord were found. A detethering of the spinal cord by sectioning the FT at L4/5 was performed (Figure 3). Intraoperatively, the FT appeared stretched, and after complete transection, cranial migration of the upper end by 1.5 cm was observed. Postoperatively, the back pain and dysesthesia resolved completely, and the patient was without symptoms at 2-year follow-up. At the age of 8 years, MRI showed an increase in the atlantoaxial distance (0.3 to 0.8 cm on flexion) with relative stenosis at the level of the foramen magnum, but no signs of compression of the medulla oblongata. Over 3 years and after a minor trauma, the horizontal instability at C1/2 increased to >1 cm, resulting in increased neck pain but no new neurologic symptoms (Figure 2, A). At the age of 12 years, navigation-guided C0-C6 spondylodesis was performed, which achieved near-complete pain relief (Figure 1, D). The risk of progressive thoracic deformity due to T3 hemivertebrae was considered high. Staged, symptom-oriented spine surgery was able to maintain neurologic integrity over several years from the onset of symptoms at early childhood.

## Discussion

CNS involvement has been described in OAVS, and frequencies range between 2% and 69%,^7^ including brain atrophy, hypoplasia of the cerebellum, corpus callosum dysgenesis, asymmetric lateral ventricles, and aqueductal stenosis with obstructive hydrocephalus, cerebral lipomas, and cerebral hamartomas.^6,7,9,10^

The pathogenesis of OAVS as well as of neural tube defects, open or closed, is attributed to disturbances in cell migration, fusion, and atrophy during the early embryonal period. OAVS has an association with spinal dysraphism of various kinds.^2,11,12,13,14^ According to Lam,^15^ a subgroup of OAVS may be a disorder of ectodermal nondisjunction early in the development with subsequent mesodermal tethering, as seen in spinal dysraphism.

But indeed, neural tube defects have been rarely reported associated with OAVS, most likely because of limited MR imaging availability. However, syringomyelia, meningocele with occipital cranium bifidum, and a defective arch of C1 as well as spina bifida cystica and occulta have been described^7,11,12,13,14,16,17^ with a prevalence of 1% to 31%.^9,10,17^

To the best of our knowledge, no case of a symptomatic occult tethered cord syndrome (OTCS) has been reported associated with OAVS. However, Brun et al^18^ described an asymptomatic low conus tip at L3 with a slightly thickened FT, with fatty infiltration in a boy with OAVS.

How a thickened and shortened FT may cause radiographic and clinical features of a tethered cord is well understood. The FT is an elastic band that stabilizes the conus medullaris. When the normal elasticity of this structure is compromised, increased tension and stress is placed on the cord, causing biochemical and electrophysiological changes that damage blood vessels, nerve fibers, and nerve cells, which is best explained by the ischemic hypothesis of Yamada et al.^19^ Ischemic changes affect the lower motor neurons, manifesting in the characteristic features of the tethered cord syndrome, which include neurologic, orthopaedic, and urogenital symptoms, most often in the presence of a low conus medullaris.^20^ But explaining symptomatic tethering of a cord ending in the normal location is more difficult, especially in cases where the FT radiographically appears almost normal, as was seen in our case (Figure 2, B). The absence of objective radiographic abnormalities but the presence of distinct clinical features of a tethered cord syndrome has raised the concept of an OTCS.^21^

As documented in our patient, the clinically suspected and intraoperatively proven tightness of the FT functionally resembles its loss of elasticity, causing symptoms of a tethered cord. Indeed, Hendson et al^22^ examined 30 fila from patients with tethered cord syndrome and histologically compared these with fila of 27 pediatric cadavers without tethered cord syndrome. The fila of all patients with tethered cord syndrome, whether the conus was low, showed abnormal fibrous connective tissue and reduction of elastic fibers in half of the patients.

Tu and Steinbok^23^ reviewed 13 retrospective case series with a total of 289 pediatric and adult patients with OTCS. Deterioration after surgery was reported in nine patients (3.28%), but urogenital function improved in 78.3%, bowel dysfunction in 88%, and pain was successfully treated in 98% of cases through surgical detethering. Cornips et al^24^ published similar results on nine pediatric patients with OTCS and radiographically normally appearing FT. All children showed improvement after detethering, similar to our patient.

Regarding the prevalence of vertebral anomalies in OAVS, we found one recent systematic review,^25^ including a large European register–based study by Barisic et al,^9^ and two more recent large retrospective cohort studies.^17,26^ The frequency of detected spine anomalies in OAVS varies from 8% to 83%. According to Caron et al,^26^ spine anomalies are markedly more frequent in patients with a more severe mandibular hypoplasia and are also markedly more often present in patients with bilateral than in patients with unilateral anomalies.

In the cervical region, various types of vertebral fusion (up to 77%), occipitalization of the atlas, odontoid hypoplasia, basilar invagination, and hemivertebrae are most often described. Besides our report, the only study mentioning cervical spine instability, by using flexion-extension radiographs, was conducted by Healey et al.^27^

Specifically, C1/C2 instability is very frequent in OAVS and may be asymptomatic in children^28^ but may progress near skeletal maturity. Healey et al^27^ reported odontoid hypoplasia with C1/C2 instability and pseudobasilar invagination in three of eight patients; Anderson and David^4^ revealed C0-C2 abnormalities (hypoplastic odontoid, assimilation of C0/1) in four of seven patients with OAVS.

In the thoracic region, hemivertebrae (10% to 50%) and semisegmented vertebrae are the most common malformations, leading to scoliosis or kyphoscoliosis in about 50%,^17,25^ as seen in our patient, in whom balanced cervicothoracic scoliosis due to a T3 hemivertebra was found.

In the lumbar region, vertebral anomalies are seen less frequently. When present, hemivertebrae, block vertebrae, and/or scoliosis are most often described. Rib anomalies (aplasia, hypoplasia, fusion, and extra ribs) in the cervical and thoracic spine have equal frequency.^17,25^

Unfortunately, in most studies, only conventional radiographs were used, so the true spectrum and extent of spine malformation cannot be delineated. Al Kaissi et al^29^ contrarily reported on anomalies in six patients with OAVS using 3D CT scans and stressed the importance of 3D scans for the proper conservative as well as surgical management of these spinal pathologies, especially in patients with syndromic associations. Two of the patients underwent surgical corrections because of a cervicothoracic scoliosis and thoracolumbar kyphosis.

Tsirikos and McMaster^30^ revealed a 2% prevalence (14 patients) of OAVS in 668 consecutive patients with congenital deformities of the spine. Eight (57%) required a spine arthrodesis. Four patients required prophylactic posterior surgery alone before the age of 2 years. However, four patients were seen at a later stage when it was necessary to perform more complex combined anterior and posterior salvage procedures. Tsirikos et al emphasize that patients with OAVS, which is usually diagnosed at birth, should be routinely referred to a spine specialist, as the identification of congenital vertebral anomalies will allow for recognition of a spinal deformity at an early stage when appropriate surgery can produce better results, reduce the risk of potential complications, and prevent unnecessary morbidity.

Because of the associated conditions, patients with OAVS frequently require surgery under general anesthesia. Owing to frequent occult craniocervical instability, but also facial-maxillary and tracheal abnormalities, intubation and spinal manipulation in these patients need specific precautions. Our case had undergone 26 surgical interventions and 19 diagnostic procedures under general anesthesia before diagnosis and surgery for C1/C2 instability. Therefore, assessment of spinal abnormalities including flexion-extension views as well as CT and MRI scans should be considered mandatory in these patients. Asymptomatic children with OAVS should be monitored by flexion-extension views at 6-month intervals and possibly be advised to minimize risk of catastrophic events by avoiding dangerous sports and activities.^27^ Spinal instrumentation/occipitocervical fusion should be considered for cases greater than 6 mm atlantoaxial distance to prevent impingement of the spinal cord. Delayed fusion in C1/2 instability may lead to progressive loss of cervical motion, requiring more complex corrections.^31,32^

## Conclusion

The prevalence and the development of spinal deformity needs to be recognized early, followed carefully, and should be treated early surgically when progressive in patients with OAVS. An OTCS in the absence of typical radiographic findings should be considered if symptoms of the conus medullaris become apparent and detethering by transection of the FT may be indicated.
